# OPA1 Mutation and Late-Onset Cardiomyopathy: Mitochondrial Dysfunction and mtDNA Instability

**DOI:** 10.1161/JAHA.112.003012

**Published:** 2012-10-25

**Authors:** Le Chen, Tingting Liu, Alice Tran, Xiyuan Lu, Alexey A. Tomilov, Vanessa Davies, Gino Cortopassi, Nipavan Chiamvimonvat, Donald M. Bers, Marcela Votruba, Anne A. Knowlton

**Affiliations:** Department of Medicine, University of California, Davis, CA (L.C., T.L., A.T., N.C., A.A.K.); Department of Pharmacology, University of California, Davis, CA (X.L., D.M.B., A.A.K.); Department of Molecular Biosciences, University of California, Davis, CA (A.A.T., G.C.); School of Optometry & Vision Sciences, Cardiff University, Cardiff, UK (V.D., M.V.); Northern California VA, Sacramento, CA (N.C., A.A.K.)

**Keywords:** cardiomyopathy, mitochondrial fusion, mtDNA, OPA1, ROS

## Abstract

**Background:**

Mitochondrial fusion protein mutations are a cause of inherited neuropathies such as Charcot–Marie–Tooth disease and dominant optic atrophy. Previously we reported that the fusion protein optic atrophy 1 (OPA1) is decreased in heart failure.

**Methods and Results:**

We investigated cardiac function, mitochondrial function, and mtDNA stability in a mouse model of the disease with OPA1 mutation. The homozygous mutation is embryonic lethal. Heterozygous OPA^+/−^ mice exhibit reduced mtDNA copy number and decreased expression of nuclear antioxidant genes at 3 to 4 months. Although initial cardiac function was normal, at 12 months the OPA1^+/−^ mouse hearts had decreased fractional shortening, cardiac output, and myocyte contraction. This coincided with the onset of blindness. In addition to small fragmented mitochondria, aged OPA1^+/−^ mice had impaired cardiac mitochondrial function compared with wild-type littermates.

**Conclusions:**

OPA1 mutation leads to deficiency in antioxidant transcripts, increased reactive oxygen species, mitochondrial dysfunction, and late-onset cardiomyopathy.

## Introduction

Mitochondria were long viewed as vital but static energy factories; however, mitochondria have been found to be dynamic organelles that not only continuously divide and fuse within the cell, but also have functions extending beyond energy production to cell signaling.^1,2^ Complete mitochondrial fusion requires 2 steps, in which the outer and inner mitochondrial membranes fuse in separate events.^2^ The mitofusins MFN1 and MFN2 are on the mitochondrial outer membrane and are involved in the first step of mitochondrial fusion, whereas optic atrophy 1 (OPA1) fuses the inner membrane. On the other hand, mitochondrial fission is controlled by Fis1 and DRP1 in mammalian cells. The rate of fission and fusion in mammalian cells is thought to be much slower than that in simpler organisms such as yeast, in which fusion and fission were first described.^3^

Recent studies have explored whether maintaining overall mitochondrial morphology and function relies on preserving the balance of fusion and fission. Imbalanced mitochondrial fusion/fission leads to apoptosis, mitochondrial DNA (mtDNA) mutations, and a severe reduction in respiratory capacity.^1,4^ There are a number of inherited neuropathies such as Charcot–Marie–Tooth disease and dominant optic atrophy that cause neuropathy and blindness. Several of these diseases have been shown to be caused by mutation of MFN2^5^ and OPA1.^6,7^ Abnormal expression of MFN2 has also been found in other diseases including Parkinson's disease and type 2 diabetes.^1^

The heart, like the brain, has a high demand for ATP and is dependent on mitochondrial high-energy phosphate production. However, only limited studies have addressed whether mitochondrial fusion/fission abnormalities have a role in heart failure. We have previously reported that OPA1^8^ is decreased in both human and rat heart failure. Electron microscopic analysis showed increased number and decreased size of mitochondria, consistent with depressed mitochondrial fusion, in a high coronary artery ligation rat heart failure model. However, it remains unknown whether this reduction of mitochondrial fusion is secondary to heart failure or a contributor to the development and progression of heart failure. Investigation has been limited on the role of OPA1-mediated mitochondrial fusion in the heart. There also remain concerns whether mitochondrial dynamic changes occur readily in cardiac muscle fibers, where mitochondria are highly organized and compacted between contractile filaments or adjacent to the sarcolemma. However, given the heart's high energy requirements, it seems very likely that changes in fusion proteins would have a negative impact on cardiac function.

To address our hypothesis that loss of fusion proteins contributes to the development of cardiomyopathy, we studied mice with an OPA1 mutation, modeling dominant optic atrophy. We found that these mice had a late onset of cardiomyopathy. Furthermore, reduction in OPA1 led to abnormalities in mitochondrial organization, depressed mitochondrial respiration, and increased mtDNA deletion, as well as increased reactive oxygen species (ROS).

## Experimental Procedures

### Mouse Breeding

OPA1 mutation has been generated as previously described; the introduced stop codon is at the beginning of the dynamin GTPase, a domain where many human disease-causing mutations cluster.^9^ OPA1^+/−^ mice and littermate controls were bred by crossing OPA1^+/−^ and WT C57Bl/6JCrl (Charles River). This has been continuing from G2 to G16 (currently). Animals aged 3 to 4 months (3 months) and 12 to 14 months (12 months) were used. All experiments were approved by the Institutional Animal Care and Use Committee at UC Davis.

### Cardiac Functional Measurements

#### Echocardiography

In all, 6 to 7 age- and sex-matched WT and OPA1^+/−^ mice were examined at different ages (monthly). Murine transthoracic echocardiography was conducted in conscious mice as previously described.^10^ Briefly, the heart was imaged in a 2-dimensional parasternal short-axis view with M-mode echocardiogram of the midventricle recorded at the level of the papillary muscle. Left ventricle (LV) fractional shortening was calculated as previously described.^10^

#### Hemodynamics measurements

Hemodynamics measurements were performed as previously described.^11^ Briefly, a lateral thoracotomy was done, and a 2.0 f Millar catheter was passed through the left carotid artery into the left ventricular chamber. Hemodynamic function was measured, including volume loops and cardiac output.

#### Cardiomyocyte contractility and calcium transients

Cardiomyocyte contractility and calcium transients were synchronously detected using an IonOptix system (IonOptix, Milton, MA) as previously described.^12^

### Histological and Electron Microscopy Analysis

The left ventricle was used for histological analysis. COX/SDH staining was performed according to the method of Chen et al.^13^ Freshly dissected heart tissue was embedded in Optimal Cutting Temperature (OCT) compound (Tissue-Tek) and frozen in liquid nitrogen. Slides were stained for COX activity, washed in water, stained for SDH activity, washed, and mounted in GelMount (Biomeda). For electron microscopy (EM), mouse hearts were fixed with 4% paraformaldehyde and then processed and imaged as described previously.^8^

### Respiration Measurements

Oxygen consumption was measured in freshly isolated cardiac mitochondria using the XF24 Seahorse instrument, modified based on a previously described method.^14^ State II respiration was initiated by adding substrate — 5 mmol/L succinate in the presence of 2 μmol/L rotenone. State III respiration was measured by the addition of ADP to a final concentration of 2.5 mmol/L. Uncoupled respiration rates were determined by injection of 2.5 μmol/L mesoxalonitrile 4-trifluoromethoxyphenylhydrazone (FCCP). State IV respiration was measured following ADP consumption. Respiration due to proton leak was determined using 0.5 μmol/L oligomycin.

Complex I, II, and IV activities were measured in freshly isolated mitochondrial lysates following the recommendations of the manufacturer (Mitoscience) as previously reported.^15^

### ATP Production Rates in Isolated Heart Mitochondria

ATP synthesis rates in isolated heart mitochondria were determined using the luciferin/luciferase-based ATP Bioluminescence Assay Kit (Sigma) essentially as described.^16^ Basal ATP content in heart left ventricle tissue was directly measured using tissue lysate. For complex I-driven ATP synthesis, 5 to 10 μg of heart mitochondria was dissolved in 50 μL of buffer A (125 KCL, 10 mmol/L HEPES, 5 mmol/L MgCl_2_, and 2 mmol/L K_2_HPO_4_, pH 7.44) plus complex I substrate pyruvate/malate, 5 mmol/L final, with and without 2 mmol/L rotenone. The ATP measurements in the presence of rotenone were subtracted to give ATP production via complex I. The measurements for all samples were started simultaneously by adding 50 μL of luciferin/luciferase buffer containing 1 mmol/L ADP (0.5 mmol/L final). Using an ATP standard provided in the kit, the slopes were converted to nanomoles per minute per milligram of protein.

### DNA Isolation

To isolate mtDNA, mouse left ventricles were first homogenized, and a mitochondrial fraction was isolated according to previously published protocols.^13^ Mitochondria were then lysed in the presence of 0.5% SDS and 0.2 mg/mL proteinase K in 10 mmol/L Tris-HCl, 0.15 mol/L NaCl, and 0.005 mol/L EDTA. mtDNA was then extracted and purified with Dynabeads (Invitrogen). Total DNA was isolated using standard protocols.

### mtDNA Quantitation

To quantify the amount of mtDNA present per nuclear genome, we used the primers as listed in Table S3. All samples were measured in triplicate, and qPCR results obtained were confirmed by 3 independent experiments. Two different primer pairs were used to quantify and confirm relative mtDNA copy number as previously described^14^: COXI and cytochrome b (mitochondrial) and β-globin/H-19 for genomic DNA. Data obtained by qPCR were analyzed by the ΔΔCT method.

### Apoptosis Assays

TUNEL staining was performed on heart sections using a commercial kit (In Situ Cell Death Detection Kit, Roche) following the manufacturer's instructions. Percentage of TUNEL positive cells was calculated as previously described.^8^

### Western Blotting

The left ventricle was homogenized in RIPA buffer as previously described,^8^ Fifty micrograms of lysate was electrophoresed and transferred to nitrocellulose membranes. Blots were blocked with 5% nonfat dry milk and incubated overnight at 4°C with anti-TFAM, PGC1-α, MFN1, MFN2, Bax, Bak, and Nrf2 antibodies (Abcam, 1:1000); GAPDH was used as an internal control. Blots then were washed and incubated with secondary antibodies (1/10 000 goat anti-rabbit-HRP/anti-mouse-HRP, GE) and developed with the Femto chemiluminescent reagent (Pierce).

### ROS Assays

ROS level was measured by a fluorescent method with confocal microscopy. CellROX deep red (Invitrogen) was used as indicator of cell ROS. Cardiomyocytes were isolated as previously described,^16^ and myocytes were incubated with CellROX at 37°C for 30 minutes. Nuclei indicator DAPI was used as a counterstain. The intensity of CellROX fluorescence was calculated and analyzed to quantify the ROS level. One hour of hypoxia followed by 1 hour of reoxygenation was performed using standard protocols as previously described.^8^ Total antioxidant capacity (TAC) was measured using a TAC ELISA kit (Cell Biolabs) following the manufacturer's protocol. Briefly, left ventricles were homogenized and centrifuged at 10 000*g* for 10 minutes at 4°C. One hundred milligrams of supernatant was loaded into a 96-well plate for subsequent TAC assay. After 30 minutes' incubation with copper ion reagent, the reduction of copper(II) to copper(I) by antioxidants was measured for absorbance at 490 nm.

### PCR Array

Total RNA was extracted from mouse left ventricle by the standard Trizol (Invitrogen) method, and PCR array was performed following the manufacturer's protocol. Mitochondrial gene and stress-related gene PCR arrays (Qiagen) were used to determine the gene profile changes in OPA1-mutant hearts. Data obtained by qPCR were analyzed by the ΔΔCT method.

### Statistical Analysis

Results are expressed as mean±SEM. Results from multiple groups were compared by analysis of variance (ANOVA) followed by a Student–Neuman–Keuls test for multiple comparisons. The Student *t* test was used for comparisons involving only 2 groups. The Wilcoxon rank-sum test and Krusakl–Wallis ANOVA were performed when data were not normally distributed. A *P*<0.05 was considered significant. In PCR array analysis, *q* value was calculated using Q-Value software (http://genomics.princeton.edu/storeylab/qvalue/). A limitation of the study was the relatively small sample size for some experiments.

## Results

### Abnormal Cardiac Function in OPA1 Mutants at 12 Months

**Figure 1.**
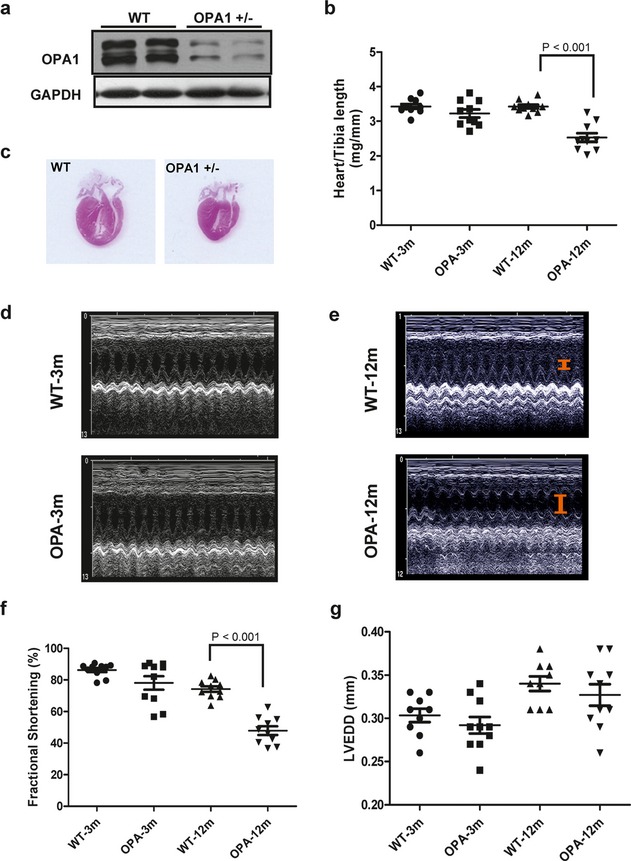
Cardiac OPA1 levels, gross cardiac morphology, and cardiac dysfunction in the OPA1^+/−^ mouse. a, OPA1 is reduced 50% in the heterozygote heart. b, Heart weight/tibia length ratio indicates the heart is slightly smaller in the mutants at 12 months (n=10 per group). c, H & E staining of representative coronal sections of the heart showing the 4 chambers from mutant and WT at 12 months. Cardiac echo from 3 to 4 months (d) and 12 months (e) mice. f, Decreased fractional shortening was evident at 12 months in the OPA1 mutants (n=10 per group). g, Left ventricular end-diastolic dimension (LVEDD, d) did not differ among groups, showing that no ventricular dilatation was present despite decline in OPA1 heterozygote heart function at 12 months (n=10 per group). WT indicates wild-type; OPA, OPA1^+/−^; 3 m, 3 to 4 months; 12 m, 12 months. Data are represented as mean±SEM, and ANOVA was used to calculate the statistical significance.

**Figure 2.**
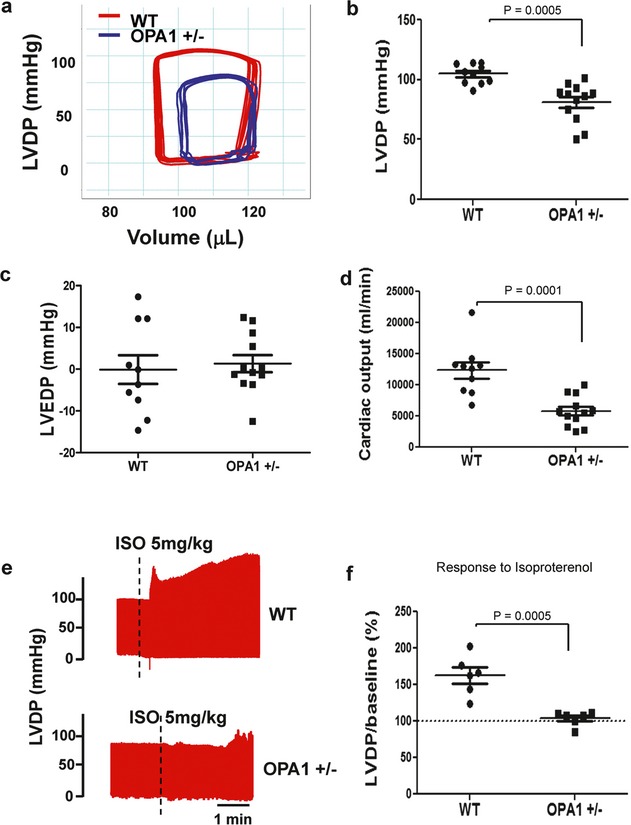
OPA1 mutation associated with impaired hemodynamics at 12 months. a, At 12 months, OPA1^+/−^ mice had abnormal pressure–volume loops compared with WT littermates. b, Left ventricular developed pressure (LVDP) was decreased at 12 months in the mutant hearts (n=10 to 12 per group). c, Cardiac output was significantly depressed at 12 months in the OPA1-mutant heart (n=10 to 12 per group). d and e, Depressed response to beta agonist – OPA1^+/−^ hearts showed markedly less response to β stimulus (isoproterenol), consistent with reduced cardiac inotropy (n=6 per group). Data are represented as mean±SEM, and the *t* test was used to calculate statistical significance.

An *OPA1* gene mutation, B6;C3-Opa1(Q285STOP), which models autosomal dominant optic atrophy, was generated in the mouse.^9^ The homozygous mutation is embryonic lethal, whereas the heterozygous mutation is associated with visual dysfunction and structural changes in the murine retina and optic nerve beginning at 12 months.^9^ The animals looked vigorous and appeared healthy. Initial descriptive studies of the heart showed no abnormalities, but specialized techniques are needed to detect many significant cardiac abnormalities. The heterozygote has a 50% reduction in the OPA1 transcript and protein in the mouse heart (Figure 1a). Given the unique arrangement of mitochondria in cardiac muscle, we examined whether OPA1 and mitochondrial fusion play an important role in this tissue. Although no significant changes in the heart weight/tibia length ratio occurred at 3 months, heart weight and chamber size were mildly decreased after 12 months in the OPA1 mutants (Figure 1b and 1c). Cardiac function was assessed monthly by echocardiogram beginning at 3 months of age in OPA1^+/−^ mice. No significant cardiac functional or gross structural abnormalities were found in these mice until 12 months, when significantly impaired contraction developed. Fractional shortening (FS) dropped from 74.18±1.81% to 47.87±2.75% (*P*<0.05) at 12 months in OPA1 mutants but did not change in WT hearts, nor in OPA1 mutants at 3 months, as shown in Figure 1d through 1f. There was no statistical difference in cardiac left ventricular end diastolic dimension (LVEDD) between the OPA1-mutant and WT groups (Figure 1g). Invasive hemodynamic studies at 12 months showed abnormal pressure–volume loops in OPA1^+/−^ mice compared with WT littermates (Figure 2a). The pressure–volume loops were mildly narrowed, consistent with decreased cardiac output in the OPA1^+/−^ hearts. Most importantly, much less pressure was generated by the OPA1^+/−^ hearts with contraction (Figure 2a). LVDPmax was significantly decreased in OPA1^+/−^ hearts compared with WT (Figure 2b), whereas there was no statistical difference in the left ventricular end diastolic pressure (LVEDP, Figure 2c). Cardiac output was markedly reduced in OPA1 mutants, from 12.37±1.87 to 5.82±0.70 mL/min (*P*<0.001), compared with WT (Figure 2d). Cardiac inotropic reserve was tested with the β-adrenergic agonist isoproterenol using a standard protocol. The 12-month OPA1^+/−^ hearts showed little to no response to β-adrenergic stimulus compared with WT (Figure 2e and 2f).

**Figure 3.**
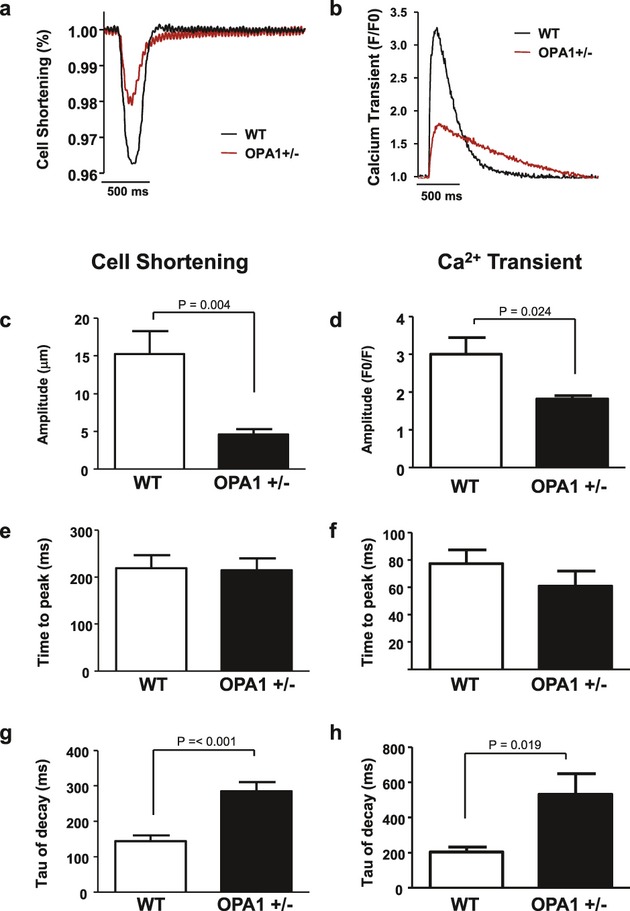
Cell shortening and calcium transients. Representative traces show depressed cell shortening (a) and calcium transients (b) in old OPA1-mutant cardiomyocytes. Amplitude, time to peak, and decay of cell shortening (c, e, and g) and calcium transients (d, f, and h) all were consistent with decreased contractility of cardiomyocytes from old OPA1 mutants. Data are represented as mean±SEM (n=9 to 10 per group). The *t* test was used to calculate statistical significance.

Calcium flux is a critical component of contractility. In isolated cardiomyocytes, 12-month OPA1 mutants had markedly reduced amplitudes of both calcium transients and contraction (Figure 3), and calcium transient decline and myocyte relaxation exhibited slowed kinetics. These changes recapitulate the cardiac dysfunction observed at the organ level and demonstrate that dysfunction at the individual myocyte level, rather than loss of myocytes per se, may be critical in overall OPA1-mutant dysfunction.

### Mitochondrial Structure and Function

#### Electron microscopy

**Figure 4.**
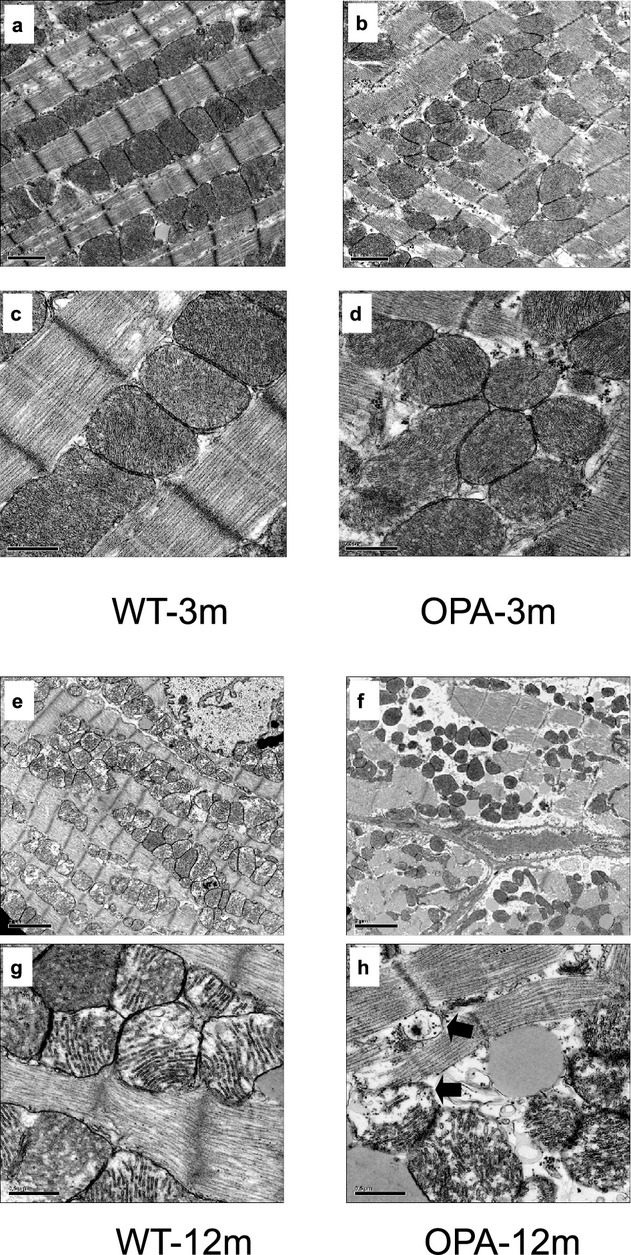
Electron microscopy of OPA1^+/−^ and WT left ventricles (a through d: 3 months; e through f: 12 months) demonstrating mitochondria losing order and separating from each other at both 3 and 12 months, which is likely to be loss of mitochondrial fusion in OPA1^+/−^ hearts (b, d, f, h) compared with WTs (a, c, e, g). Higher-magnitude images show mitochondria with loss of the normal mitochondrial cristae structure (indicated by arrows) at 12 months in OPA1 mutants (h) compared with WTs (g), suggested incomplete mitochondrial respiratory function. WT indicates wild type; OPA, OPA1^+/−^; 3 m, 3 to 4 months; 12 m, 12 months.

EM was used to investigate cellular structure in the left ventricle (Figure 4). The young WT heart showed orderly arrays of mitochondria between myofilaments (Figure 4a and 4c). This pattern was not found in OPA1^+/−^ hearts (Figure 4b and 4d). The OPA1^+/−^ hearts had greater disruption of mitochondrial organization along with decreased density of mitochondria/area, suggesting of loss of mitochondria (Figure 4c and 4d). Higher magnification (Figure 4g and 4h) revealed damage to the normal mitochondrial cristae structure with loss of cristae, consistent with impaired mitochondrial respiratory function. The WT cardiac mitochondria showed normal alignment along the sarcomere, but there was some loss of cristae at 12 months (Figure 4e and 4g), likely from aging.^17^

#### Mitochondrial function

**Figure 5.**
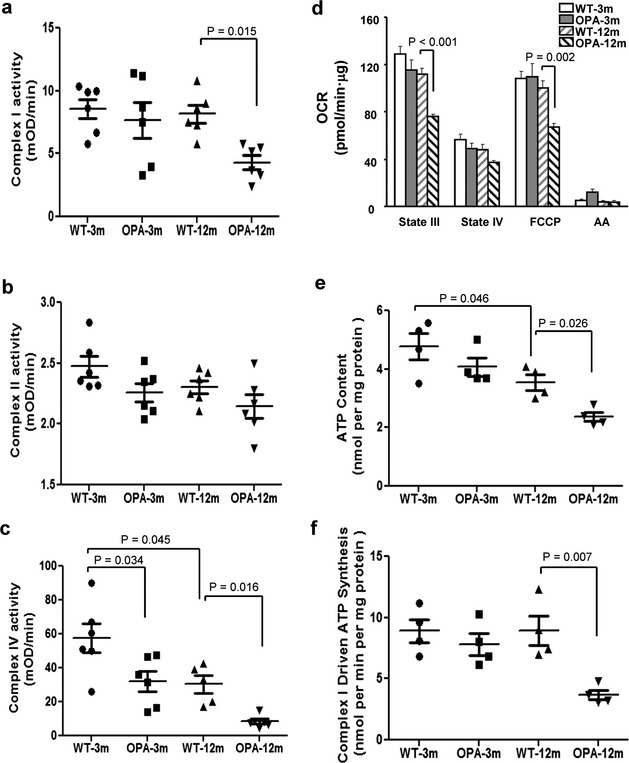
Mitochondrial dysfunction in OPA1-mutant hearts. a through c, Enzyme activities of complex I, II, and IV measured in WT and OPA1^+/−^ mouse hearts (n=6 to 7 per group). d, Oxygen consumption rates (OCR) in WT and OPA1^+/−^ heart mitochondria in the presence of succinate. State III was induced by injection of ADP. State IV was induced by inhibition of ATP synthase with oligomycin, and uncoupled respiration rates were determined by injection of mesoxalonitrile 4-trifluoromethoxyphenylhydrazone (FCCP). Antimycin A (AA) was used to determine background, nonmitochondrial OXPHOS, OCR (n=7 to 8 per group). e, Basal ATP content (n=4 per group). f, Complex I–driven ATP synthesis measured in freshly isolated cardiac mitochondria in the presence of the complex I substrate pyruvate/malate, with and without rotenone. The ATP measurements in the presence of rotenone were subtracted to give ATP production via complex I. WT (n=4 per group) indicates wild type; OPA, OPA1^+/−^; 3 m, 3 to 4 months; 12 m, 12 to 14 months. Data are represented as mean±SEM, and ANOVA was used to calculate statistical significance.

Enzyme activities for complexes I, II, and IV were measured in vitro in WT and OPA1^+/−^ mouse hearts. Fresh cardiac mitochondrial lysates were prepared and complex enzyme activities measured using a 96-well plate–based assay (Mitosciences, Eugene, OR). Activities of complexes I and IV decreased significantly in OPA1^+/−^ hearts, whereas complex II activity remained unchanged compared with WT (Figure 5a through 5c). Direct mitochondrial respiration studies using freshly isolated mitochondria from OPA1^+/−^ and WT left ventricles were done to complement the in vitro assays of complex function. Depressed oxygen consumption was detected both in succinate/ADP-driven state III respiration and in FCCP-induced maximal respiration capacity in the 12-month OPA1^+/−^ cardiac mitochondria, whereas no difference was found in the young (Figure 5d). Consistent with these findings, basal cardiac ATP content in left ventricular tissue lysate was significantly decreased in the aged OPA1^+/−^ compared with aged WT hearts (*P*<0.05). A more modest but significant decrease in ATP occurred in the aged WT (P<0.05; Figure 5e). Complex I–driven ATP synthesis was measured in freshly isolated cardiac mitochondria, and there was a significant decrease in ATP synthesis in aged OPA1^+/−^ hearts, whereas no statistical differences were found in the young groups (*P*<0.01; Figure 5f). These results suggest that OPA1 plays a critical role in maintaining mitochondrial respiration and sustaining mitochondrial energy production at 12 months, which are known to be defective in heart failure.

### Mitochondrial DNA

**Figure 6.**
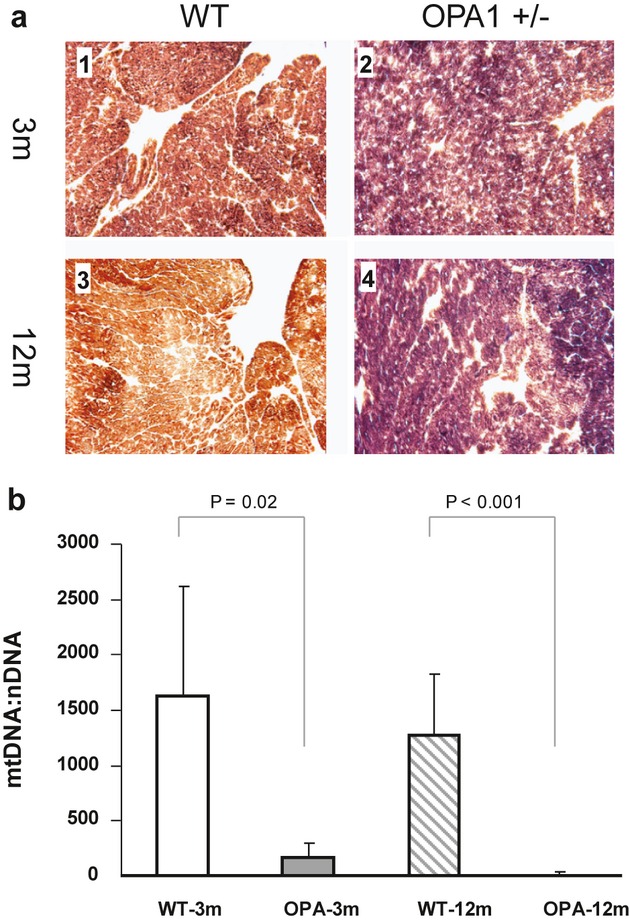
Loss of mtDNA stability in OPA1-mutant hearts. a, Transverse sections of the left ventricle were stained for COX (brown) and SDH (blue) activity. Blue staining indicates mitochondrial dysfunction (reduction in COX) and mtDNA deletion. b, Quantitative analysis of cardiac mtDNA copy number per nuclear genome at 3 and 12 months (n=4 to 5 per group). WT indicates wild type; OPA, OPA1^+/−^; 3 m, 3 to 4 months; 12 m, 12 to 14 months. Data are represented as mean±SEM, and ANOVA was used to calculate statistical significance.

Given the ultrastructural and functional evidence for mitochondrial dysfunction, we first assessed mitochondrial-encoded versus nuclear-encoded mitochondrial protein activity using traditional histochemical staining for cytochrome c oxidase (COX, complex IV, brown stain) and succinate dehydrogenase (SDH, complex II, blue stain). In both 3- and 12-month wild-type mice, the cardiac sections showed predominantly brown COX staining of the cardiac fibers (Figure 6a, 1 and 3). However, in OPA1-mutant hearts, the staining pattern in both groups was predominantly blue, indicating reduced COX activity (Figure 6a, 2 and 4). This characteristic abnormal blue histological pattern is often found in cases of respiratory dysfunction due to mtDNA defects,^13,18^ because SDH activity is encoded solely by the nuclear genome, whereas COX activity is dependent on the mitochondrial genome. The decreased COX staining is consistent with the reduction of mitochondrial complex IV enzyme activity in the OPA1-mutant hearts (Figure 5c).

**Figure 7.**
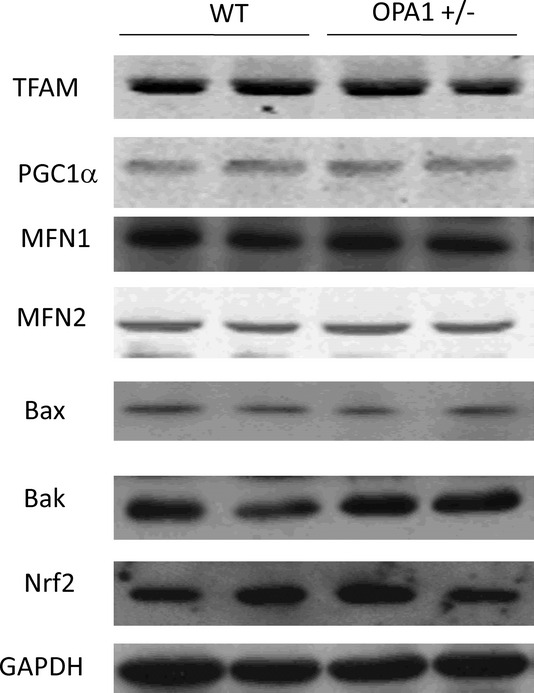
Expression of key proteins in OPA1^+/−^ vs WT hearts at 12 months. Western blots showing unchanged TFAM, PGC1-α, MFN1, MFN2, Bax, Bak, and Nrf2 in OPA1-mutant hearts. GAPDH was used as a loading control.

Real-time PCR was employed to more precisely investigate mtDNA copy number using the approach described by Sahin et al.^14^ Despite the lack of grossly abnormal pathology or function for the young OPA1 hearts, mtDNA copy number was markedly reduced compared with WT littermates (Figure 6b). The OPA1-mutant hearts had even greater reduction in mtDNA copies per nuclear genome at 12 months (Figure 6b). The mtDNA loss in this case is not likely related to mitochondrial biogenesis because PGC-1α and TFAM protein content was unchanged (Figure 7).

### OPA1 Mutation and Oxidative Stress

**Figure 8.**
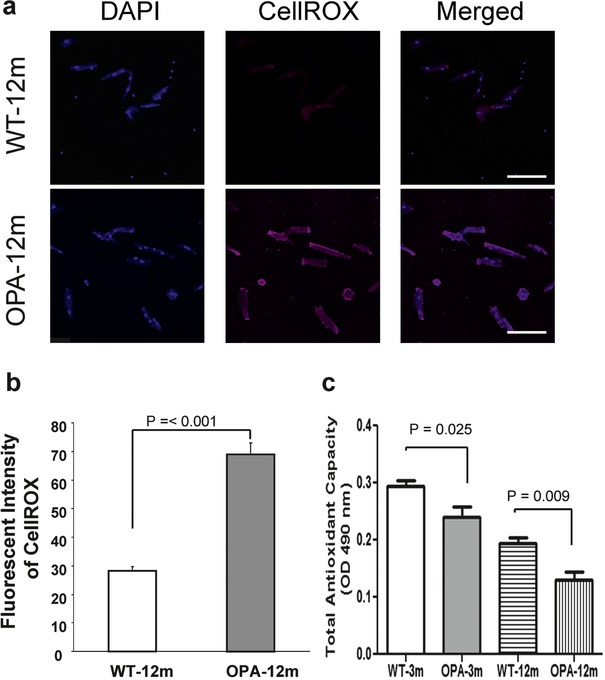
Reactive oxygen species (ROS) accumulated in OPA1-mutant hearts. a, Confocal microscopy image showing ROS accumulated in 12-month OPA1-mutant cardiomyocytes (n=4 donors per group). ROS was detected by a fluorogenic probe, CellROX Deep Red reagent. Nuclei stained with DAPI. Bar=100 μm. b, Summary of ROS intensity data from multiple experiments. c, Total antioxidant capacity measured in WT and OPA1-mutant left ventricles (n=5 per group). WT indicates wild type; OPA, OPA1^+/−^; 3 m, 3 to 4 months; 12 m, 12 to 14 months. Data are represented as mean±SEM, and the *t* test was used to calculate statistical significance.

**Figure 9.**
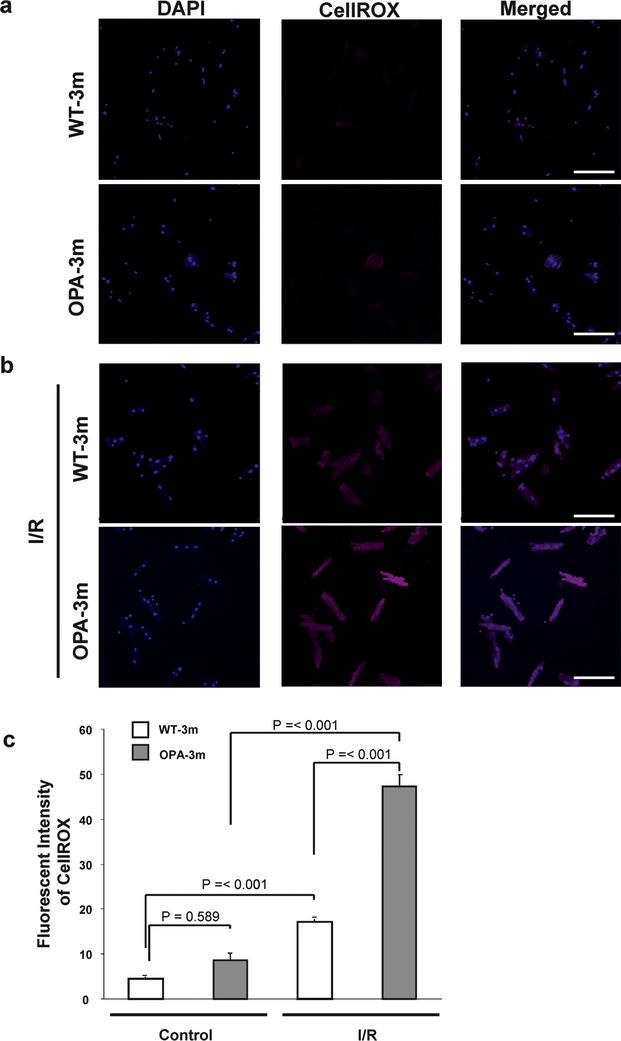
Reactive oxygen species (ROS) measured in young OPA1-mutant cardiomyocytes. a, ROS are undetectable in either WT or OPA1^+/−^ cardiomyocytes in the basal state. b, ROS after 1 hour of simulated ischemia with 1 hour of reoxygenation. c, Summary of ROS intensity data from multiple experiments. ROS were detected by a fluorogenic probe, CellROX Deep Red reagent (n=4 donors per group). Nuclei were stained with DAPI. Bar=100 μm. WT indicates wild type; OPA, OPA1^+/−^; 3 m: 3 to 4 months; I/R, ischemia/reoxygenation. Data are represented as mean±SEM, and ANOVA was used to calculate statistical significance.

**Figure 10.**
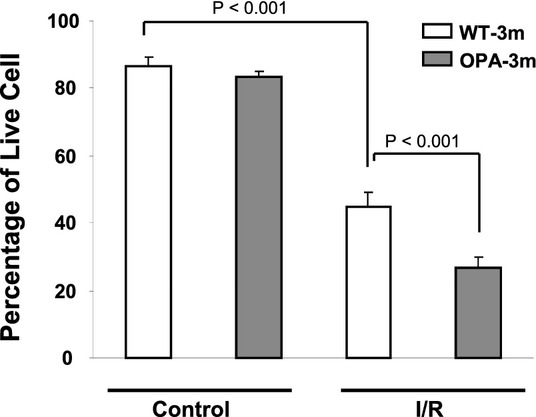
Cardiomyocyte viability after simulated ischemia followed by reoxygenation. Decreased OPA^+/−^-cardiomyocyte viability after 6 hours of simulated ischemia and 1 hour of reoxygenation measured by a Live/Dead Viability Kit, in which green-fluorescent calcein-AM indicates live cells and red-fluorescent ethidium homodimer-1 indicates dead cells with loss of plasma membrane integrity. Percentage of live cardiomyocytes was calculated (n=4 donors per group). WT indicates wild type; OPA, OPA1^+/−^; 3 m, 3 to 4 months; I/R, ischemia/reoxygenation. Data are represented as mean±SEM, and ANOVA was used to calculate statistical significance.

Studies of ROS in OPA1-mutant cardiomyocytes showed increased resting ROS compared with WT controls (Figure 8a and 8b) at 12 months, indicating reduced ability to handle ROS in OPA1-mutant hearts. A PCR array focused on oxidative stress-related genes demonstrated a marked reduction in the number of antioxidant genes, both at young and older ages, in the OPA1-mutant heart (Table 1, the entire list can be found as Table S1). Downregulated antioxidant enzymes included Gpx3 and Gstk1. In the young OPA1-mutant heart, the DNA repair-related gene *Ercc6* and the oxidative stress mediator protein Txnip were upregulated. These results suggest that OPA1 mutants may be more vulnerable to ROS-inducing factors such as ischemia/reperfusion. At 3 months, both OPA1-mutant and WT myocytes showed low basal ROS levels. After 1 hour of hypoxia and 1 hour of reoxygenation, both mutant and WT young myocytes had increased ROS levels, but levels from the mutant myocytes were strikingly increased compared with WT myocytes (Figure 9). Moreover, by using a cell Live/Dead assay (Invitrogen), it was found that 6 hours of hypoxia and 1 hour of reoxygenation induced significantly more cell death in young OPA1-mutant cardiomyocytes compared with WT myocytes (*P*<0.001, Figure 10), indicating greater vulnerability to ROS stress in the OPA1-mutant heart. In addition, total antioxidant capacity was reduced significantly in both young and older OPA1-mutant hearts compared with WT hearts (Figure 8c).

**Table 1.** Summary of Transcriptional Regulation of Oxidative Stress–Related Genes

Gene	Description	Function	Fold Induction	*P*
WT vs OPA1^+/−^ (3 to 4 months)				
*Ercc6*	Excision repair cross-complementing rodent repair deficiency, complementation group 6	DNA repair	1.88	0.044

*Txnip*	Thioredoxin interacting protein	Oxidative stress mediator	2.29	0.028

*Gpx2*	Glutathione peroxidase 2	Antioxidants	−3.34	0.014

*Gpx3*	Glutathione peroxidase 3	Antioxidants	−3.10	0.038

*Gstk1*	Glutathione S-transferase kappa 1	Antioxidants	−2.16	0.038

*Txnrd2*	Thioredoxin reductase 2	Antioxidants	−5.01	0.047

WT vs OPA1^+/−^ (12 months)				

*Cyba*	Cytochrome b-245, alpha polypeptide	Antioxidants	−1.31	0.028

*Gpx3*	Glutathione peroxidase 3	Antioxidants	−2.67	0.007

*Gstk1*	Glutathione S-transferase kappa 1	Antioxidants	−1.63	0.024

*Prdx6-rs1*	Peroxiredoxin 6, related sequence 1	Antioxidants	−3.41	0.021

*Srxn1*	Sulfiredoxin 1 homolog (*Saccharomyces cerevisiae*)	Antioxidants	−2.63	0.039

Positive value indicates upregulation; negative value, downregulation; WT, wild type; and OPA1, optic atrophy 1.

### OPA1 Mutation and Apoptosis

**Figure 11.**
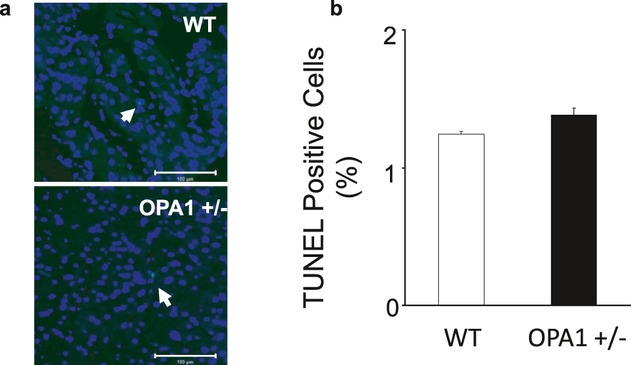
Apoptosis at 12 months. a, Representative confocal images of TUNEL-positive cells in left ventricle sections by TUNEL. Green fluorescence indicates TUNEL-positive apoptotic nuclei, with DAPI as a nuclear counter stain. b, Percentage of apoptotic cardiomyocytes was calculated based on TUNEL-positive fluorescence of cardiac myocytes/total cardiac myocytes (n=3 donors per group). Data are represented as mean±SEM, and the *t* test was used to calculate statistical significance.

Although OPA1-mediated mitochondrial fusion has been linked to protection from apoptosis,^19^ there was no statistical difference in TUNEL-positive cells or histological evidence of cardiac myocyte loss in OPA1-mutant hearts (Figure 11). There were no statistical differences found in mitochondrial intrinsic apoptosis-related genes, including *Bak1, Bcl2, Bcl2l1,* and *Bnip3* (Table S2). In addition, no difference was detected in the proapoptotic Bak and Bax proteins (Figure 7).

## Discussion

Homozygous null mutations of OPA1 are embryonic lethal.^9^ The heterozygote had slow development of cardiomyopathy, characterized by reduced fractional shortening, markedly reduced inotropy, abnormal calcium transients, mitochondrial dysfunction leading to reduced ATP levels, decreased antioxidant gene expression, and increased ROS. However, despite these many changes, we detected no increase in apoptosis. mtDNA copy number was strikingly reduced, and this certainly contributed to some of the abnormalities, including complex IV dysfunction through reduction of COX expression. Interestingly, the onset of eye disease^9^ and cardiomyopathy occurred simultaneously at 12 months. These finding suggest a progressive pathological process induced by OPA1 reduction. Although there are some changes with aging in the WT, leading to mild reduction in ATP content and mitochondrial ultrastructure, the OPA1 mutant has far more dramatic effects on mitochondrial morphology and function. Recently, Dorn et al reported that silencing of OPA1 and mitochondrial assembly regulatory factor (MARF) induces heart tube dilation in a *Drosophila* model.^20^ In another report, a different OPA1 splicing mutation, which leads to an in-frame deletion of 27 amino acid residues in the GTPase domain, showed no evidence of cardiac dysfunction at 6 months but surprisingly demonstrated more severe hypertrophy after chronic pressure overload.^21^ Furthermore, it has been reported that MFN2-deficient mice display modest cardiac hypertrophy accompanied by slight functional deterioration.^22^ Reduction in either MFN1 or MFN2 can be partially compensated for by the other MFN protein, suggesting some overlap in function.^23^ Knockout of either MFN1 or MFN2 was protective against ischemia/reperfusion injury in cardiomyocytes,^22,24^ whereas a combination knockout of MFN1/2 led to rapidly lethal cardiac failure.^25^ The mitochondrial fission protein Drp1 has been linked to dilated cardiomyopathy.^26^ OPA1 is the only protein known to mediate inner mitochondrial membrane fusion. Our previous work demonstrated a significant decrease of OPA1 protein content in both human and rat heart failure, whereas MFN1/2 surprisingly increased in human heart failure.^8^ In the current study, OPA1 mutation led to cardiomyopathy without changing the gene and protein level of MFN1/2 (Table S2 and Figure 7). These results suggest a unique role of OPA1 in regulating mitochondrial fusion, and depressed mitochondrial fusion may play a critical role in the downward progression of cardiomyopathies.

Decreased ATP production in OPA1-mutant hearts is likely a major factor in the decreased function, as demonstrated by echocardiograhy and hemodynamics. Depressed cardiomyocyte contractility and calcium transients may also partly result from the depressed ATP levels. To further understand the mechanism linking OPA1 to ATP generation, we measured mitochondrial oxygen consumption in the OPA1-mutant hearts. OCR decreased significantly in the OPA1-mutant cardiac mitochondria at 12 months. This is not surprising, as there are reports demonstrating a direct correlation between mitochondrial fusion and oxidative phosphorylation capacity.^27^ Inhibition of mitofusins-mediated mitochondrial fusion in skeletal muscle,^13^ fibroblasts,^28^ and MEFs^23^ results in reduced oxygen consumption. The depressed OCR suggested electron transport chain (ETC) dysfunction, and this was confirmed by in vitro enzyme activity assays demonstrating that complex I and complex IV had decreased activity, whereas complex II activity was normal. This result is consistent with our finding of decreased COX activity (complex IV) by staining.

Although the pancreas-specific knockout of OPA1 does not affect mtDNA copy number,^29^ our results demonstrate convincing loss of mtDNA copies in both young and older OPA1-mutant hearts. There is one theory that mitochondrial fusion/fission serves as a means of distribution of mtDNA to the progeny of mammalian cells,^30^ which is supported by experiments demonstrating that the loss of mitofusins leads to loss of mtDNA in muscle.^13^ Recently, Elachouri et al found that silencing of OPA1 leads to mtDNA depletion, secondary to inhibition of mtDNA replication.^31^ In a series of clinical studies, specific OPA1 mutations were observed to induce the accumulation of mtDNA deletions in the skeletal muscle of patients.^32,33^ Altogether these observations suggested that altering mitochondrial dynamics affects the maintenance of mtDNA integrity. The loss of mtDNA induced by OPA1 mutation may be the key factor in the development of cardiac mitochondrial dysfunction.

The mitochondrial fusion proteins OPA1 and MFN1/2 have been shown to protect cells from apoptosis. Increased fission, decreased fusion, or both can induce caspase activation, Bax translocation to mitochondria, and cytochrome c release.^19^ Bak may also regulate apoptosis by interacting with the mitofusins.^34^ Interestingly, Bax may also positively regulate mitochondrial fusion exclusively through homotypic MFN2 trans complexes.^35^ However, most of these results were generated in cultured cells. Even though our data from a myocytelike cell line indicates reduction of OPA1 induces apoptosis,^8^ we did not detect an increase in TUNEL-positive apoptotic myocytes in mouse left ventricular sections. Furthermore, we did not find changes in Bax or Bak expression in OPA1-mutant hearts. Thus, the mechanism of decreased cardiac function in the OPA1 mutant is not an increase in apoptosis. A similar conclusion was reached in an in vivo study in the pancreas,^29^ where no increase in apoptosis associated with pancreas-specific knockout of OPA1.

OPA1 mutation–related cardiomyopathy was associated with increased ROS generation. Decreased mitochondrial ETC complex activity and mtDNA depletion may have contributed to this ROS accumulation. The accumulated ROS could also damage the ETC and mtDNA, creating a vicious cycle of damage. We were surprised to find marked depression of expression of a number of nuclear-encoded antioxidant genes, which would further exacerbate the ROS damage. The protein content of Nrf2, a transcription factor inducing expression of several antioxidant enzymes, was unchanged in OPA1-mutant hearts (Figure 7). The link between reduced OPA1 and reduced nuclear transcription will need to be addressed in future studies. Although there is no marked cardiac dysfunction evident in OPA1 mutants at 3 to 4 months, the decreased mtDNA copy number and increased ROS accumulation in these functionally “normal” hearts are evidence of significant abnormality despite normal function.

Mitochondrial morphologic abnormalities are associated with different types of cardiomyopathy^8,36^ in which OPA1 may be involved. Further work will be needed to fully define the role of abnormal mitochondrial fission/fusion in heart failure. The contribution of unbalanced mitochondrial fusion/fission to heart failure as a cause of myocardial injury remains to be proven. Nevertheless, the restoration of mitochondrial fusion/fission symmetry may help to rescue the failing heart. Treatment of adult murine cardiac myocytes with mitochondrial division inhibitor-1, a pharmacological inhibitor of Drp1, reduced cell death and infarct size after ischemia/reperfusion injury.^37^ No cardiac disorders have been described in patients with OPA1 or similar mutations involving the fission/fusion genes as seen in inherited maladies like Charcot–Marie–Tooth disease. Our results indicate that, at least for OPA1, cardiac abnormalities are not completely manifest until the development of blindness. Although the OPA1-mutant mice survived more than 1 year and appeared healthy, we cannot exclude the changes in other organs had an effect on cardiac function, though this is unlikely. Thus, in patients with these diseases, reduced cardiac function may go undetected secondary to reduced physical activity secondary to loss of vision. It would be expected that patients with such mutations would have impaired cardiac reserve with reduced ability to respond to high-stress disease states such as myocardial infarction and sepsis. The OPA1-mutant mice have reduced cardiac reserve, as shown by the lack of response to isoproterenol or to ischemia/reperfusion injury, as shown in the current study, suggesting that patients with OPA1 and related inherited mitochondrial diseases should be screened for abnormalities of cardiac function.

## References

[1] Liesa M Palacin M Zorzano A Mitochondrial dynamics in mammalian health and disease. Physiol Rev. 2009;89:799-845. 19584314 10.1152/physrev.00030.2008

[2] Chen H Chan DC Physiological functions of mitochondrial fusion. Ann NY Acad Sci. 2010;1201:21-25. 20649534 10.1111/j.1749-6632.2010.05615.x

[3] Legros F Lombès A Frachon P Rojo M Mitochondrial fusion in human cells is efficient, requires the inner membrane potential, and is mediated by mitofusins. Mol Biol Cell. 2002;13:4343-4354. 12475957 10.1091/mbc.E02-06-0330 PMC138638

[4] Chen H Chan DC Mitochondrial dynamics – fusion, fission, movement, and mitophagy – in neurodegenerative diseases. Hum Mol Genet. 2009;18:R169-R176. 19808793 10.1093/hmg/ddp326 PMC2758711

[5] Zuchner S Mersiyanova IV Muglia M Bissar-Tadmouri N Rochelle J Dadali EL Zappia M Nelis E Patitucci A Senderek J Parman Y Evgrafov O Jonghe PD Takahashi Y Tsuji S Pericak-Vance MA Quattrone A Battaloglu E Polyakov AV Timmerman V Schroder JM Vance JM Mutations in the mitochondrial GTPase mitofusin 2 cause Charcot-Marie-Tooth neuropathy type 2A. Nat Genet. 2004;36:449-451. 15064763 10.1038/ng1341

[6] Alexander C Votruba M Pesch U Thiselton D Mayer S Moore A Rodriguez M Kellner U Leo-Kottler B Auburger G Bhattacharya S Wissinger B OPA1, encoding a dynamin-related GTPase, is mutated in autosomal dominant optic atrophy linked to chromosome 3q28. Nat Genet. 2000;26:211-215. 11017080 10.1038/79944

[7] Delettre C Lenaers G Griffoin JM Gigarel N Lorenzo C Belenguer P Pelloquin L Grosgeorge J Turc-Carel C Perret E Astarie-Dequeker C Lasquellec L Arnaud B Ducommun B Kaplan J Hamel CP Nuclear gene OPA1, encoding a mitochondrial dynamin-related protein, is mutated in dominant optic atrophy. Nat Genet. 2000;26:207-210. 11017079 10.1038/79936

[8] Chen L Gong Q Stice JP Knowlton AA Mitochondrial OPA1, apoptosis, and heart failure. Cardiovasc Res. 2009;84:91-99. 19493956 10.1093/cvr/cvp181 PMC2741347

[9] Davies VJ Hollins AJ Piechota MJ Yip W Davies JR White KE Nicols PP Boulton ME Votruba M OPA1 deficiency in a mouse model of autosomal dominant optic atrophy impairs mitochondrial morphology, optic nerve structure and visual function. Hum Mol Genet. 2007;16:1307-1318. 17428816 10.1093/hmg/ddm079

[10] Xu D Li N He Y Timofeyev V Lu L Tsai H-J Kim I-H Tuteja D Mateo RKP Singapuri A Davis BB Low R Hammock BD Chiamvimonvat N Prevention and reversal of cardiac hypertrophy by soluble epoxide hydrolase inhibitors. Proc Natl Acad Sci USA. 2006;103:18733-18738. 17130447 10.1073/pnas.0609158103 PMC1693731

[11] Kohout TA Takaoka H McDonald PH Perry SJ Mao L Lefkowitz RJ Rockman HA Augmentation of cardiac contractility mediated by the human β3-adrenergic receptor overexpressed in the hearts of transgenic mice. Circulation. 2001;104:2485-2491. 11705829 10.1161/hc4501.098933

[12] Lu X-Y Chen L Cai X-L Yang H-T Overexpression of heat shock protein 27 protects against ischaemia/reperfusion-induced cardiac dysfunction via stabilization of troponin I and T. Cardiovasc Res. 2008;79:500-508. 18397962 10.1093/cvr/cvn091

[13] Chen H Vermulst M Wang YE Chomyn A Prolla TA McCaffery JM Chan DC Mitochondrial fusion is required for mtDNA stability in skeletal muscle and tolerance of mtDNA mutations. Cell. 2010;141:280-289. 20403324 10.1016/j.cell.2010.02.026 PMC2876819

[14] Sahin E Colla S Liesa M Moslehi J Muller FL Guo M Cooper M Kotton D Fabian AJ Walkey C Maser RS Tonon G Foerster F Xiong R Wang YA Shukla SA Jaskelioff M Martin ES Heffernan TP Protopopov A Ivanova E Mahoney JE Kost-Alimova M Perry SR Bronson R Liao R Mulligan R Shirihai OS Chin L DePinho RA Telomere dysfunction induces metabolic and mitochondrial compromise. Nature. 2011;470:359-365. 21307849 10.1038/nature09787 PMC3741661

[15] Li Q Vande Velde C Israelson A Xie J Bailey AO Dong M-Q Chun S-J Roy T Winer L Yates JR Capaldi RA Cleveland DW Miller TM ALS-linked mutant superoxide dismutase 1 (SOD1) alters mitochondrial protein composition and decreases protein import. Proc Natl Acad Sci USA. 2010;107:21146-21151. 21078990 10.1073/pnas.1014862107 PMC3000256

[16] Gao H Chen L Yang H-T Activation of α1b-adrenoceptors alleviates ischemia/reperfusion injury by limitation of mitochondrial Ca2+ overload in cardiomyocytes. Cardiovasc Res. 2007;75:584-595. 17509549 10.1016/j.cardiores.2007.04.008

[17] Kelly DP Cell biology: ageing theories unified. Nature. 2011;470:342-343. 21307852 10.1038/nature09896

[18] Reeve AK Krishnan KJ Turnbull D Mitochondrial DNA mutations in disease, aging, and neurodegeneration. Ann NY Acad Sci. 2008;1147:21-29. 19076427 10.1196/annals.1427.016

[19] Lee YJ Jeong SY Karbowski M Smith CL Youle RJ Roles of the mammalian mitochondrial fission and fusion mediators Fis1, Drp1, and Opa1 in apoptosis. Mol Biol Cell. 2004;15:5001-5011. 15356267 10.1091/mbc.E04-04-0294 PMC524759

[20] Dorn GW II Clark CF Eschenbacher WH Kang MY Engelhard JT Warner SJ Matkovich SJ Jowdy CC MARF and Opa1 control mitochondrial and cardiac function in Drosophila. Circ Res. 2011;108:12-17. 21148429 10.1161/CIRCRESAHA.110.236745 PMC3337031

[21] Piquereau J Caffin F Novotova M Prola A Garnier A Mateo P Fortin D Huynh LH Nicolas V Alavi MV Brenner C Ventura-Clapier R Veksler V Joubert F Down-regulation of OPA1 alters mouse mitochondrial morphology, PTP function, and cardiac adaptation to pressure overload. Cardiovasc Res. 2012;94:408-417. 22406748 10.1093/cvr/cvs117 PMC3863708

[22] Papanicolaou KN Khairallah RJ Ngoh GA Chikando A Luptak I O'Shea KM Riley DD Lugus JJ Colucci WS Lederer WJ Stanley WC Walsh K Mitofusin-2 maintains mitochondrial structure and contributes to stress-induced permeability transition in cardiac myocytes. Mol Cell Biol. 2011;31:1309-1328. 21245373 10.1128/MCB.00911-10 PMC3067905

[23] Chen H Chomyn A Chan DC Disruption of fusion results in mitochondrial heterogeneity and dysfunction. J Biol Chem. 2005;280:26185-26192. 15899901 10.1074/jbc.M503062200

[24] Papanicolaou KN Ngoh GA Dabkowski ER O'Connell KA Ribeiro RF Stanley WC Walsh K Cardiomyocyte deletion of mitofusin-1 leads to mitochondrial fragmentation and improves tolerance to ROS-induced mitochondrial dysfunction and cell death. Am J Physiol Heart Circ Physiol. 2012;302:H167-H179. 22037195 10.1152/ajpheart.00833.2011 PMC3334239

[25] Chen Y Liu Y Dorn GW II Mitochondrial fusion is essential for organelle function and cardiac homeostasis. Circ Res. 2011;109:1327-1331. 22052916 10.1161/CIRCRESAHA.111.258723 PMC3237902

[26] Ashrafian H Docherty L Leo V Towlson C Neilan M Steeples V Lygate CA Hough T Townsend S Williams D Wells S Norris D Glyn-Jones S Land J Barbaric I Lalanne Z Denny P Szumska D Bhattacharya S Griffin JL Hargreaves I Fernandez-Fuentes N Cheeseman M Watkins H Dear TN A mutation in the mitochondrial fission gene Dnm1l leads to cardiomyopathy. PLoS Genet. 2010;6:e1001000 20585624 10.1371/journal.pgen.1001000 PMC2891719

[27] Zanna C Ghelli A Porcelli AM Karbowski M Youle RJ Schimpf S Wissinger B Pinti M Cossarizza A Vidoni S Valentino ML Rugolo M Carelli V OPA1 mutations associated with dominant optic atrophy impair oxidative phosphorylation and mitochondrial fusion. Brain. 2008;131:352-367. 18222991 10.1093/brain/awm335

[28] Bach D Pich S Soriano FX Vega N Baumgartner B Oriola J Daugaard JR Lloberas J Camps M Zierath JR Rabasa-Lhoret R Wallberg-Henriksson H Laville M Palacin M Vidal H Rivera F Brand M Zorzano A Mitofusin-2 determines mitochondrial network architecture and mitochondrial metabolism. J Biol Chem. 2003;278:17190-17197. 12598526 10.1074/jbc.M212754200

[29] Zhang Z Wakabayashi N Wakabayashi J Tamura Y Song W-J Sereda S Clerc P Polster BM Aja SM Pletnikov MV Kensler TW Shirihai OS Iijima M Hussain MA Sesaki H The dynamin-related GTPase OPA1 is required for glucose-stimulated ATP production in pancreatic beta cells. Mol Biol Cell. 2011;22:2235-2245. 21551073 10.1091/mbc.E10-12-0933 PMC3128526

[30] Margineantu DH Gregory Cox W Sundell L Sherwood SW Beechem JM Capaldi RA Cell cycle dependent morphology changes and associated mitochondrial DNA redistribution in mitochondria of human cell lines. Mitochondrion. 2002;1:425-435. 16120295 10.1016/s1567-7249(02)00006-5

[31] Elachouri G Vidoni S Zanna C Pattyn A Boukhaddaoui H Gaget K Yu-Wai-Man P Gasparre G Sarzi E Delettre C Olichon A Loiseau D Reynier P Chinnery PF Rotig A Carelli V Hamel CP Rugolo M Lenaers G OPA1 links human mitochondrial genome maintenance to mtDNA replication and distribution. Genome Res. 2011;21:12-20. 20974897 10.1101/gr.108696.110 PMC3012919

[32] Hudson G Amati-Bonneau P Blakely EL Stewart JD He L Schaefer AM Griffiths PG Ahlqvist K Suomalainen A Reynier P McFarland R Turnbull DM Chinnery PF Taylor RW Mutation of OPA1 causes dominant optic atrophy with external ophthalmoplegia, ataxia, deafness and multiple mitochondrial DNA deletions: a novel disorder of mtDNA maintenance. Brain. 2008;131:329-337. 18065439 10.1093/brain/awm272

[33] Amati-Bonneau P Valentino ML Reynier P Gallardo ME Bornstein B Boissiere A Campos Y Rivera H de la Aleja JG Carroccia R Iommarini L Labauge P Figarella-Branger D Marcorelles P Furby A Beauvais K Letournel F Liguori R La Morgia C Montagna P Liguori M Zanna C Rugolo M Cossarizza A Wissinger B Verny C Schwarzenbacher R Martin MA Arenas J Ayuso C Garesse R Lenaers G Bonneau D Carelli V Opa1 mutations induce mitochondrial DNA instability and optic atrophy ‘plus’ phenotypes. Brain. 2008;131:338-351. 18158317 10.1093/brain/awm298

[34] Brooks C Wei Q Feng L Dong G Tao Y Mei L Xie ZJ Dong Z Bak regulates mitochondrial morphology and pathology during apoptosis by interacting with mitofusins. Proc Natl Acad Sci USA. 2007;104:11649-11654. 17606912 10.1073/pnas.0703976104 PMC1913853

[35] Hoppins S Edlich F Cleland MM Banerjee S McCaffery JM Youle RJ Nunnari J The soluble form of Bax regulates mitochondrial fusion via MFN2 homotypic complexes. Mol Cell. 2011;41:150-160. 21255726 10.1016/j.molcel.2010.11.030 PMC3072068

[36] Hom J Yu T Yoon Y Porter G Sheu SS Regulation of mitochondrial fission by intracellular Ca2+ in rat ventricular myocytes. Biochim Biophys Acta. 2010;1797:913-921. 20347716 10.1016/j.bbabio.2010.03.018 PMC2891135

[37] Ong SB Subrayan S Lim SY Yellon DM Davidson SM Hausenloy DJ Inhibiting mitochondrial fission protects the heart against ischemia/reperfusion injury. Circulation. 2010;121:2012-2022. 20421521 10.1161/CIRCULATIONAHA.109.906610

